# Impact of probiotics and prebiotics on glucose/lipid metabolism in metabolic dysfunction-associated steatotic liver disease: mechanisms and implications

**DOI:** 10.3389/fnut.2026.1779954

**Published:** 2026-05-08

**Authors:** Yinan Zhao, Ziyan Li, Guoying Yu

**Affiliations:** 1 Clinical Medical College of Qinghai University, Xining, China; 2 Department of Hepatology II, Fourth People’s Hospital of Qinghai Province, Xining, China

**Keywords:** functional foods, glucose metabolism, gut-liver axis, insulin resistance, lipid metabolism, MASLD, prebiotics, probiotics

## Abstract

Metabolic dysfunction-associated steatotic liver disease (MASLD) is a growing global health concern, intricately linked to metabolic disorders such as obesity, dyslipidemia, and insulin resistance. As conventional pharmacological treatments often show limited efficacy, there is increasing interest in functional foods, particularly probiotics and prebiotics, as potential therapeutic strategies for managing MASLD and related metabolic disturbances. This perspective article explores the mechanisms by which probiotics and prebiotics influence glucose and lipid metabolism in MASLD. And by synthesizing data from recent high-level meta-analyses and umbrella reviews (up to 2025),provides a quantitative mini-meta-summary of current clinical evidence. Probiotics modulate the gut-liver axis, reducing inflammation and improving lipid metabolism, while prebiotics, such as dietary fibers and bioactive compounds, promote beneficial gut microbiota changes that support metabolic regulation. Both preclinical and clinical studies provide evidence of their beneficial effects on liver function, insulin sensitivity, and lipid profiles. This article bridges the gap between mechanistic insights and clinical outcomes, emphasizing the transition from generic supplementation to evidence-based, duration-targeted therapeutic strategies. Future research should focus on identifying optimal probiotic strains and prebiotic compounds, investigating their synergistic effects, and conducting large-scale clinical trials to solidify their therapeutic potential.

## 1 Introduction

Metabolic dysfunction-associated steatotic liver disease (MASLD), formerly known as non-alcoholic fatty liver disease (NAFLD), has emerged as the most common chronic liver disorder worldwide and a critical component of the global metabolic disease pandemic (1, 2). MASLD is defined by the presence of hepatic steatosis in conjunction with metabolic dysfunction, including obesity, insulin resistance, dyslipidemia, or type 2 diabetes (3, 4). Recent epidemiological evidence indicates that MASLD currently affects approximately 38% of the global adult population, with prevalence steadily increasing over the past decades as obesity and metabolic disorders become more widespread (5). By 2040, adult MASLD prevalence is projected to exceed 55% globally if current trends persist (5).

The burden of MASLD is heterogeneous across different populations (6). Individuals with metabolic risk factors, notably those with type 2 diabetes, demonstrate particularly elevated prevalence rates, with over 65% of diabetic individuals affected, and face an increased risk of progression to more severe disease phenotypes (6–8). Furthermore, MASLD significantly contributes to liver-related morbidity and mortality, as well as extra-hepatic complications such as cardiovascular disease and chronic kidney disease, thereby exacerbating the global public health challenge (9, 10).

Despite this alarming epidemiological profile, effective pharmacological options remain limited, and lifestyle modifications alone have shown variable efficacy (11, 12). As a result, there has been growing interest in functional foods, particularly probiotics and prebiotics, as complementary strategies to modulate metabolic pathways, improve gut-liver axis homeostasis, and ultimately mitigate the metabolic derangements underpinning MASLD (11, 13). Early preclinical and clinical studies suggest that probiotics and prebiotics may beneficially influence lipid metabolism, glucose regulation, and low-grade inflammation, but pivotal questions remain about their mechanisms and clinical application (14, 15).

In this perspective, we address four key questions that frame current research and future directions:

1. How do probiotics and prebiotics contribute to the modulation of inflammation and metabolic pathways in MASLD?
2. What are the specific mechanisms through which probiotics and prebiotics influence glucose and lipid metabolism in MASLD?
3. What evidence do preclinical studies provide regarding the efficacy of probiotics and prebiotics as interventions for MASLD?
4. What are the main challenges and limitations in advancing the translation of probiotics and prebiotics from experimental research to clinical practice in MASLD?

**FIGURE 1:**
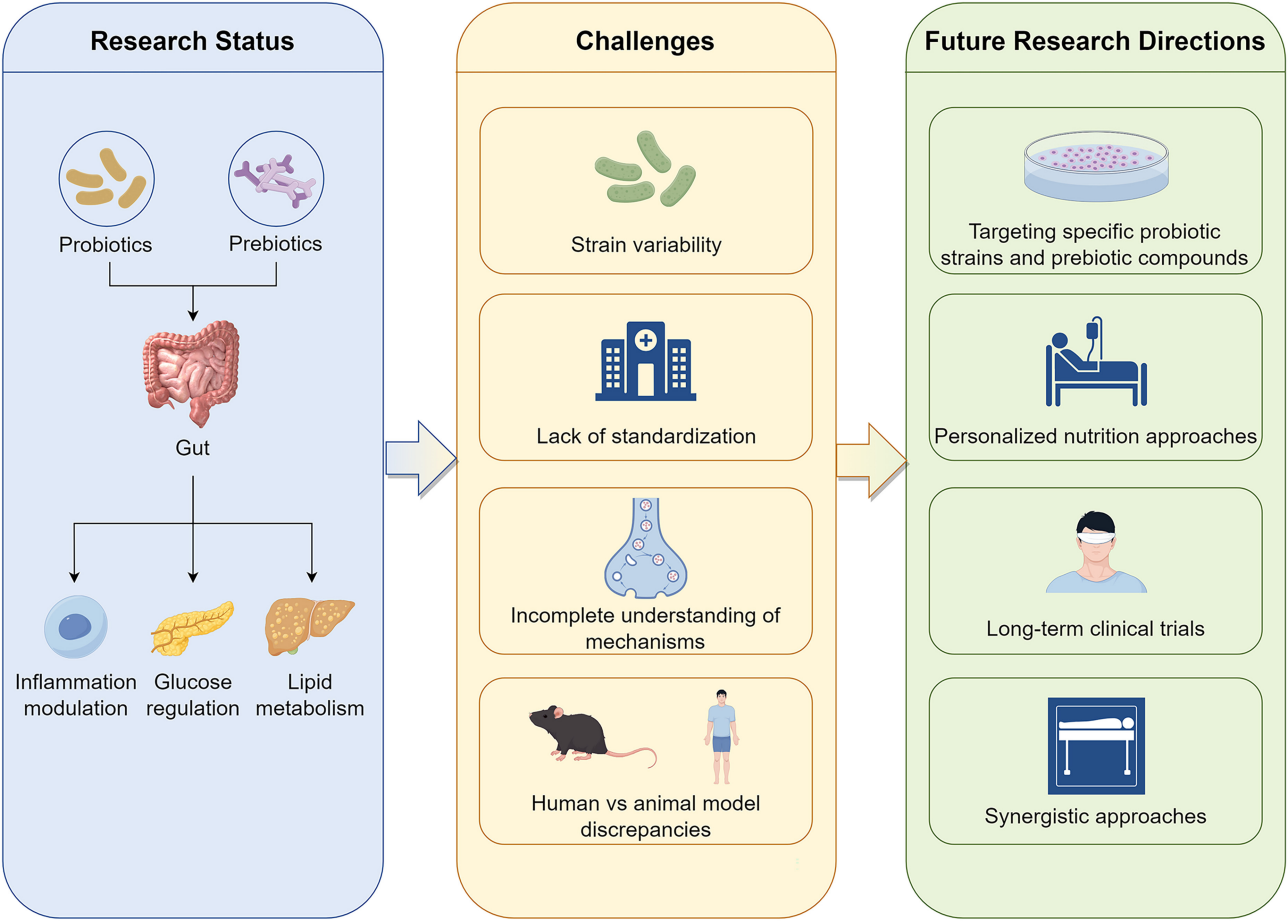
Current research landscape, challenges, and future directions in MASLD. The left panel depicts current mechanistic insights where these interventions modulate the gut-liver axis to improve lipid metabolism and glucose homeostasis while reducing systemic inflammation. Moving to the center, the Figure highlights critical bottlenecks hindering clinical translation, including probiotic strain variability, the lack of standardization in dosage and protocols, an incomplete understanding of molecular mechanisms, and translational discrepancies between animal models and human trials. Finally, the right panel proposes key strategies for advancement such as identifying targeted specific strains and bioactive compounds, adopting microbiome-based personalized nutrition approaches, conducting long-term clinical trials to validate efficacy, and exploring synergistic therapies combined with conventional treatments.

Figure 1 illustrates the current research landscape, challenges, and future research directions regarding the role of probiotics and prebiotics in MASLD. It highlights the impact of these functional foods, the challenges in their application, and the potential avenues for further research.

## 2 Mechanisms of probiotics and prebiotics in MASLD

**FIGURE 2:**
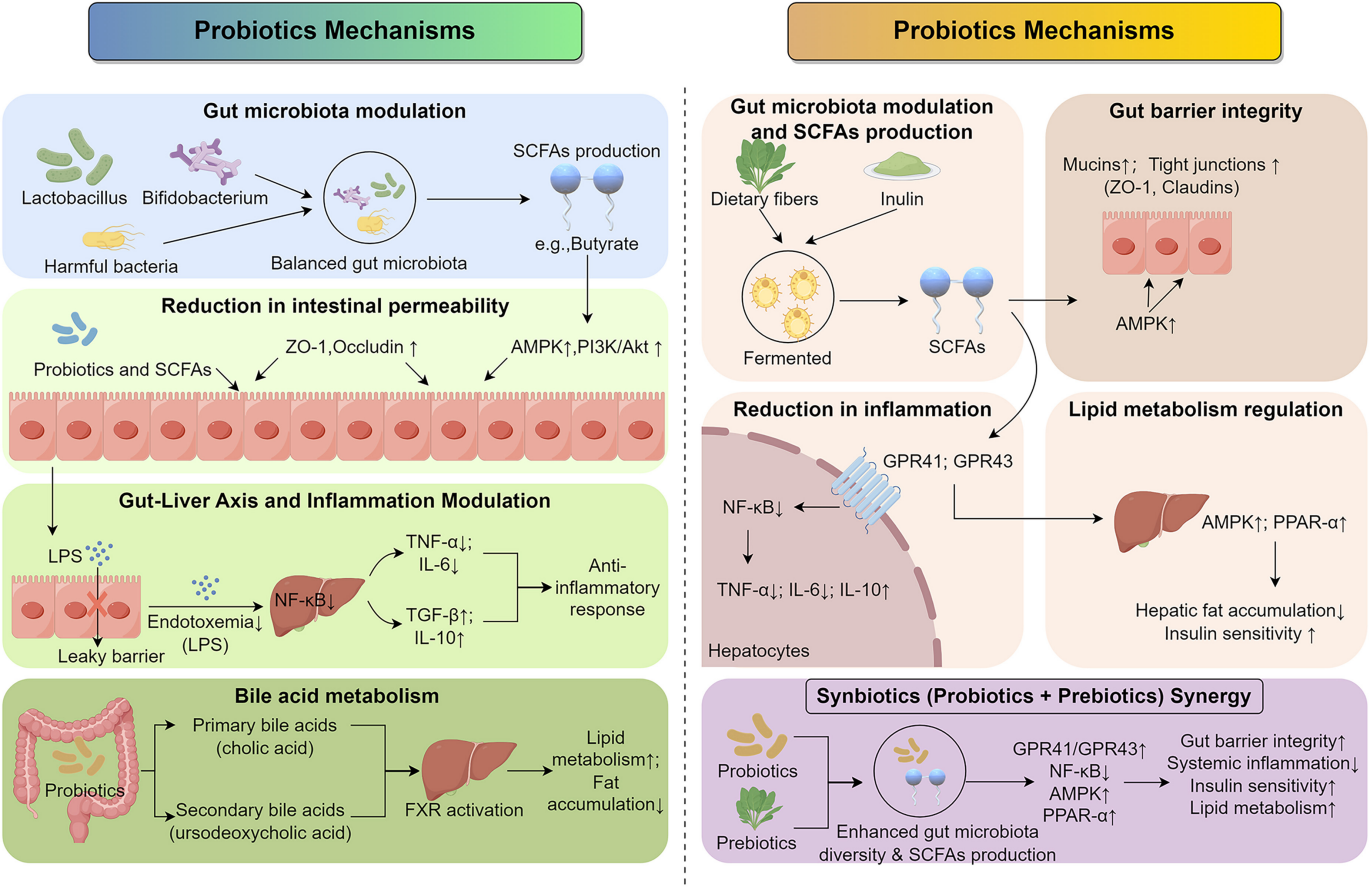
Mechanisms of Probiotics and Prebiotics in MASLD. Probiotics and prebiotics modulate the gut-liver axis through distinct yet complementary pathways. Probiotics reduce dysbiosis and strengthen the gut barrier (ZO-1, Occludin) to limit endotoxemia (LPS) and hepatic inflammation (NF-κB pathway). Prebiotics undergo fermentation to produce SCFAs, which activate GPR41/43 signaling to improve insulin sensitivity and lipid metabolism (AMPK, PPAR-α pathways). Synbiotics combine these strategies to exert synergistic effects on systemic inflammation and metabolic homeostasis.

The pathogenesis of MASLD is closely linked to the disruption of metabolic and immune processes in the liver, often driven by systemic inflammation, insulin resistance, and dysbiosis of the gut microbiota (16, 17). Probiotics and prebiotics have emerged as potential therapeutic agents due to their ability to modulate these processes and restore metabolic balance (18, 19). The mechanisms by which these functional foods exert their effects are complex and multifaceted, involving interactions between the gut, liver, and immune system (see Figure 2).

Probiotics, defined as live microorganisms that confer a health benefit to the host when administered in adequate amounts, have been shown to improve MASLD outcomes through several mechanisms (13, 20). One of the primary ways probiotics affect MASLD is by modulating the gut microbiota (21). Dysbiosis, or an imbalance in the gut microbial community, is frequently observed in individuals with MASLD and is associated with increased intestinal permeability, which leads to the translocation of endotoxins such as lipopolysaccharides (LPS) into the bloodstream (22, 23). This activates inflammatory pathways and contributes to liver injury (22). Probiotics can restore gut microbiota balance, reduce intestinal permeability, and subsequently lower endotoxin levels, thereby reducing systemic inflammation and liver injury (24). Moreover, certain probiotic strains have been found to influence bile acid metabolism, which is crucial for lipid homeostasis (25). By modulating the composition of bile acids, probiotics can reduce hepatic fat accumulation and improve lipid profiles (24, 25). Additionally, probiotics may exert anti-inflammatory effects by regulating the production of cytokines and immune cells in the liver, thereby improving insulin sensitivity and reducing hepatic steatosis (24, 26).

Prebiotics, which are non-digestible food components that selectively stimulate the growth or activity of beneficial gut microbes, have been shown to support metabolic health by enhancing gut microbiota diversity and promoting the production of short-chain fatty acids (SCFAs) such as butyrate (27, 28). SCFAs play a significant role in regulating glucose metabolism, lipid metabolism, and inflammation (27, 28). Butyrate, in particular, has been shown to reduce liver fat accumulation and improve insulin sensitivity by modulating gene expression in the liver and muscle tissue (29, 30). Butyrate, a product of prebiotic fermentation, has been shown to enhance gut barrier integrity by stimulating the production of mucins and tight junction proteins. This enhancement serves as a protective mechanism, preventing the translocation of harmful substances into the bloodstream. (31). This reduction in intestinal permeability can decrease systemic inflammation and improve liver function (32). Additionally, prebiotics may stimulate the release of gut hormones such as glucagon-like peptide-1 (GLP-1), which is involved in regulating insulin secretion and glucose homeostasis (27).

The combination of probiotics and prebiotics, known as synbiotics, may offer enhanced benefits for MASLD by providing both microbial support and dietary components that foster a healthy gut environment (11, 33). Synbiotics have been shown to synergistically enhance gut microbiota diversity, improve gut barrier function, and optimize metabolic regulation (34). The synergistic effects of probiotics and prebiotics may also offer a more robust anti-inflammatory response, as both work in tandem to modulate immune cell activity and reduce pro-inflammatory cytokine production in the liver (35). Together, probiotics and prebiotics address multiple aspects of MASLD pathogenesis, including dysbiosis, inflammation, insulin resistance, and lipid accumulation (36, 37). Understanding the specific mechanisms through which these functional foods operate will be crucial for optimizing their use in clinical practice and for the development of targeted therapeutic strategies for MASLD.

## 3 Clinical evidence on probiotics and prebiotics in MASLD

The clinical application of probiotics and prebiotics in managing MASLD has garnered increasing attention. Several randomized controlled trials (RCTs) have assessed the effects of these interventions on liver function, particularly focusing on key outcomes like alanine aminotransferase (ALT) reductions and steatosis scores. Probiotic treatments have demonstrated significant improvements in liver function in patients with metabolic disorders. A comprehensive meta-analysis of 12 RCTs including 657 participants revealed that probiotic supplementation significantly reduced liver damage markers, including ALT by 8.12 IU/L (95% CI: [−12.00, −4.24]; *p* < 0.0001), aspartate aminotransferase (AST) by 7.98 IU/L, and GGT by 6.43 IU/L (13). One key study by Behrouz et al. reported that daily probiotic intake led to a significant reduction in ALT and AST levels, particularly in patients suffering from both MASLD and metabolic syndrome, highlighting the potential of probiotics in managing liver inflammation and injury (15). Furthermore, recent findings suggest that the magnitude of clinical benefits is closely associated with intervention duration, with peak efficacy typically observed between 12 and 24 weeks (38).

Beyond liver enzymes, microbial therapy shows substantial benefits in lipid profiles and hepatic morphology. A meta-analysis by Yao et al. reviewed the impact of probiotic supplementation on liver enzymes, showing a moderate reduction in ALT levels across studies (11). An umbrella review of 23 meta-analyses involving 18,999 patients confirmed that probiotics are most effective in reducing total cholesterol (TC), while prebiotics show superior effects in reducing triglycerides (TG) (11). Synbiotics, on the other hand, appear most effective in reducing LDL-C and improving insulin resistance as measured by HOMA-IR (11). Prebiotics, particularly fiber-rich compounds like inulin and fructooligosaccharides, have also been explored for their therapeutic role in MASLD. A trial by Kurban et al. demonstrated that prebiotic supplementation improved hepatic steatosis and reduced systemic inflammation, with participants showing lower levels of inflammatory cytokines like TNF-α and CRP (12). Umbrella meta-analysis of 14 studies demonstrated that microbial therapy significantly improves hepatic steatosis with an Odds Ratio (OR) of 2.612 (95% CI: [1.674, 4.075]; *p* < 0.001) and leads to a reduction in liver stiffness measurement (LSM) by an effect size of −0.550 (12).

Additionally, the combination of probiotics and prebiotics, known as synbiotics, has shown promising synergistic effects in clinical settings (12). Lee et al. found that synbiotics led to significant improvements in both liver function and metabolic health, including reductions in ALT and liver fat accumulation (39). Synbiotics also contributed to better gut microbiota balance, which is essential in the regulation of systemic inflammation and insulin sensitivity, making them a potentially valuable intervention for MASLD (39). These findings suggesting that prebiotics can be an effective adjunct in managing liver health in individuals with MASLD. Overall, the growing body of clinical evidence supports the potential of probiotics and prebiotics as therapeutic strategies in the management of MASLD, though further research is needed to optimize treatment protocols and determine long-term benefits.

## 4 Mini-meta-summary of quantitative clinical improvements

Current evidence from umbrella reviews and large-scale meta-analyses underscores the systemic metabolic benefits of “biotics” in MASLD patients (see Table 1). Probiotics/prebiotics significantly reduce ALT (MD: −8.12 IU/L), AST (MD: −7.98 IU/L), and TC (MD: −7.91 mg/dL) (13). Most importantly, microbial interventions demonstrate a high probability of resolving hepatic steatosis (OR: 2.612) and show a preliminary capacity to alleviate hepatic fibrosis (ES: −0.274) (12). While pediatric patients show more consistent improvements in steatosis remission—reaching up to 91% remission in specific trials (40)—adult populations tend to exhibit larger reductions in lipid parameters (13). These findings suggest that microbial therapy is a promising adjunctive strategy to standard lifestyle modifications.

**TABLE 1:** Quantitative pooled effects of microbial therapy on MASLD clinical outcomes.

Outcome domain	Clinical indicator	Pooled effect size (MD / OR / ES)	95% confidence interval (CI)	Statistical significance (*p*)	References
Liver morphology	Hepatic steatosis resolution (OR)	2.612	[1.674, 4.075]	<0.001	(12)
	Liver stiffness (LSM, ES)	−0.55	[−0.716, −0.384]	<0.001	
	Hepatic fibrosis degree (ES)	−0.274	[−0.427, −0.120]	<0.001	
Liver injury	ALT (IU/L)	−8.12	[−12.00, −4.24]	<0.0001	(13)
	AST (IU/L)	−7.98	N/A	<0.001	
	GGT (IU/L)	−6.43	N/A	<0.0001	
Lipid profile	Triglycerides (TG, mg/dL)	−9.35	[−16.36, −2.34]	0.009	(13)
	Total cholesterol (TC, mg/dL)	−7.91	[−9.76, −6.06]	<0.0001	
	LDL-C (mg/dL)	−6.31	[−7.64, −4.98]	<0.00001	
	HDL-C (mg/dL)	+3.31	[0.24, 6.02]	0.03	
Metabolic state	BMI (kg/m^2^)	−1.08	N/A	<0.05	(11)
	Plasma glucose (mg/dL)	−4.45	N/A	<0.001	

Lee et al. found that for MASLD patients, 12 weeks of multi-strain synbiotics combined with Fructooligosaccharides led to significant improvements in lipid profiles (TC and LDL-C), FBS, and liver function (ALT/AST), alongside a reduction in systemic inflammation markers like TNF-α and IL-6 (39). Overall, the growing body of clinical evidence supports the potential of probiotics and prebiotics as therapeutic strategies in the management of MASLD, though further research is needed to determine the optimal strain-specific protocols and long-term benefits.

## 5 Preclinical evidence on probiotics and prebiotics in MASLD

The therapeutic potential of probiotics and prebiotics in MASLD has been increasingly explored through preclinical studies. These studies provide valuable insights into the efficacy and mechanisms through which probiotics and prebiotics may alleviate the metabolic disturbances associated with MASLD, such as hepatic steatosis, insulin resistance, and dyslipidemia. Preclinical studies, particularly those involving animal models, have contributed significantly to our understanding of the mechanisms by which probiotics and prebiotics influence MASLD pathogenesis (see Table 2). These studies have demonstrated promising results, though challenges remain in translating these findings to human populations.

**TABLE 2:** Preclinical evidence on probiotics and prebiotics in MASLD.

Intervention	Model	Key findings	Reference
*Lactobacillus rhamnosus* GG	C57BL/6J mice; high-fat diet-induced MASLD	*Lactobacillus rhamnosus* GG reduced intestinal fatty acid absorption, decreased weight gain and hepatic lipid accumulation in HFD-fed mice.	(42)
*Lactiplantibacillus plantarum* BD7807	C57BL/6J mice; high-fat diet-induced metabolic disorders	BD7807 improved lipid metabolism, intestinal barrier function, and reduced inflammation by modulating SCFAs-GPR43 pathway.	(59)
Inulin	Sprague-Dawley rats; high-sucrose diet-induced MASLD	Inulin improved hepatic steatosis and inflammation, and restored regulators of hepatic lipogenesis.	(51)
Fructooligosaccharides + Probiotics	C57BL/6J mice; western diet-induced MASLD	Fructooligosaccharides and probiotics significantly improved weight gain, liver enlargement, and inflammatory markers.	(39)

Numerous studies have investigated the impact of various probiotic strains on metabolic dysfunctions associated with MASLD in animal models, particularly those dysfunctions induced by high-fat diets or other obesity-related conditions (18, 41). For example, the administration of *Lactobacillus rhamnosus* and *Bifidobacterium longum* has demonstrated a reduction in hepatic fat accumulation and an enhancement of insulin sensitivity in rodents subjected to high-fat diets (42, 43). These advantageous effects are attributed to the probiotics’ capacity to restore gut microbiota equilibrium, diminish systemic inflammation, and modulate bile acid metabolism (13, 44, 45). Furthermore, *Lactobacillus plantarum* has been observed to improve lipid profiles and decrease oxidative stress in the liver, thereby alleviating hepatic damage (46). Probiotics such as Lactobacillus have also been shown to reduce the expression of pro-inflammatory cytokines (TNF-α, IL-6) and increase anti-inflammatory cytokines (IL-10) in liver tissue, suggesting a key role for probiotics in modulating inflammation (47). In models of metabolic dysfunction-associated steatohepatitis (MASH), probiotics have been associated with a reduction in hepatic fibrosis and improved liver function markers, including ALT and AST (48).

Prebiotics, primarily dietary fibers and oligosaccharides, have also shown promising effects in animal models of MASLD (49, 50). Inulin, a soluble fiber, has been particularly well-studied for its beneficial effects on gut microbiota composition and metabolic health (51, 52). Animal studies have demonstrated that inulin supplementation reduces hepatic lipid accumulation and improves glucose tolerance in rodents with diet-induced obesity (51). The effects of inulin are largely mediated through its ability to stimulate the growth of beneficial gut microbes, leading to increased production of SCFAs, particularly butyrate, which has anti-inflammatory and insulin-sensitizing properties (53). Similarly, fructooligosaccharides and galactooligosaccharides have been shown to enhance gut microbiota diversity and promote the growth of beneficial bacteria such as Bifidobacterium and Lactobacillus (54, 55). These prebiotics also improved liver function, reduced fat deposition, and ameliorated dyslipidemia in rodent models (49). Furthermore, prebiotics have been shown to upregulate the expression of genes involved in lipid oxidation, contributing to improved metabolic efficiency in the liver (51).

The concurrent administration of probiotics and prebiotics, collectively referred to as synbiotics, has been assessed across various animal models, demonstrating favorable outcomes (56). Synbiotics have been observed to more effectively mitigate hepatic fat accumulation, enhance insulin sensitivity, and restore the diversity of gut microbiota (36, 57). In mouse models of MASLD, synbiotics have led to significant reductions in serum cholesterol levels and hepatic triglycerides (58). These findings underscore the synergistic potential of probiotics and prebiotics in the management of MASLD.

Animal studies have indicated potential therapeutic benefits of probiotics and prebiotics in the management of MASLD; however, several limitations persist. The variability in probiotic strains, prebiotic types, and dosages utilized across different studies poses challenges in establishing optimal treatment protocols. Moreover, the majority of these animal studies are characterized by small sample sizes and short-term interventions, which inadequately address the long-term efficacy and individual adaptability of probiotics and prebiotics in MASLD treatment. Consequently, there is an urgent need for rigorously designed, large-scale, and long-term clinical trials to thoroughly assess the efficacy of probiotics, prebiotics, and synbiotics in MASLD management. Such trials should incorporate multicenter, randomized controlled designs and systematically evaluate various probiotic strains, prebiotic types, and dosages to ascertain the most effective treatment regimen. Additionally, these studies should prioritize the investigation of long-term treatment outcomes, safety profiles, differential efficacy across diverse populations, and the synergistic effects of combining these interventions with conventional treatments.

## 6 Challenges and future directions

Although probiotics and prebiotics have shown considerable promise in preclinical and clinical studies for managing MASLD, several challenges and limitations must be addressed before these interventions can be fully integrated into clinical practice. One of the primary challenges in the use of probiotics for MASLD treatment is the significant variability in the effects of different probiotic strains (60). The efficacy of probiotics is highly strain-dependent, and not all strains provide the same therapeutic benefits (24, 60). For example, some strains may have strong anti-inflammatory effects, while others may primarily impact gut microbiota composition or bile acid metabolism (24, 60). This variability makes it difficult to determine which probiotic strains are most effective for treating MASLD. Future studies should focus on isolating and testing specific probiotic strains that have demonstrated promising results in preclinical models, particularly those that reduce liver fat accumulation, improve insulin sensitivity, and modulate inflammatory pathways (61). Similarly, the selection of prebiotics requires further validation through additional experimental studies. While prebiotics such as inulin and fructooligosaccharides have shown benefits, but more research is necessary to evaluate other prebiotics that may be more effective in promoting gut health and metabolic regulation (62, 63).

A notable challenge in the field is the absence of standardization concerning the types and dosages of prebiotics and probiotics employed in preclinical and clinical studies. Variability in formulations, dosages, and delivery methods across different studies complicates the comparison of results and the formulation of definitive conclusions (64). For example, probiotic dosages can differ significantly between studies, with concentrations ranging from low to high (10^8^ to 10^11^ CFU), and the efficacy of these varying doses is not consistently established (65). This lack of consistency highlights the urgent need for rigorously designed clinical trials that adhere to standardized protocols regarding the type, strain, dosage, and duration of treatment (35). Standardizing the duration of clinical trials to at least 3 months is critical for capturing maximum efficacy, as clinical benefits often peak between 8 and 24 weeks (38). Pharmacological treatments, such as those targeting insulin resistance and lipid metabolism, are often used alongside lifestyle interventions (11, 12). Moreover, investigating how probiotics and prebiotics can complement these treatments could lead to more comprehensive therapeutic strategies, potentially improving patient outcomes (14, 66).

Despite progress in elucidating the mechanisms by which probiotics and prebiotics affect MASLD, the precise molecular pathways remain incompletely understood. Their observed effects—modulating gut microbiota, reducing inflammation, and improving lipid metabolism—are complex and multifactorial (26, 67). A comprehensive understanding of their interactions with specific metabolic and immune pathways is essential for optimizing therapeutic application (11). Research on the gut-liver axis and the relevant signaling pathways is still nascent, and bridging this knowledge gap is critical for developing targeted therapies.

Translating preclinical efficacy to human populations remains a major hurdle (68, 69). Animal models often fail to replicate human disease complexity, and interspecies differences in immune responses, metabolism, and gut microbiota can lead to discrepant outcomes (70). Moreover, most animal studies are short-term and controlled, whereas human trials involve diverse variables such as diet, lifestyle, and genetics (70). Personalizing probiotic/prebiotic treatments based on individual gut microbiota and genetic profiles may enhance efficacy (40, 71). Future research should develop tools to profile gut microbiota and identify predictive biomarkers (72). Incorporating personalized factors can improve outcomes and minimize side effects (11). Multi-center trials in diverse populations are needed to assess long-term effects and interactions with other treatments (14).

Future research should also explore the synergistic effects and optimal treatment durations of combining probiotics and prebiotics with conventional therapies for MASLD (35, 66). Investigating how probiotics and prebiotics can complement pharmacological treatments targeting insulin resistance or lipid metabolism could lead to more comprehensive therapeutic strategies (14, 66). Bridging the knowledge gap in molecular pathways and addressing discrepancies between animal models and human trials remain essential hurdles for translational success. The concept of “Precision Biotics”—tailoring treatments based on an individual’s gut microbiota composition—could lead to more effective and targeted interventions (40).

## 7 Discussion

The growing evidence supporting the use of probiotics and prebiotics in managing MASLD highlights their potential as complementary therapeutic strategies. Probiotics and prebiotics work through multiple mechanisms, including gut microbiota modulation, reduction of intestinal permeability, and improvement in metabolic and inflammatory pathways, all of which are critical in the pathogenesis of MASLD. Despite promising preclinical findings, several challenges remain, including strain variability, lack of standardization, and incomplete understanding of the underlying molecular mechanisms.

Our qualitative and quantitative synthesis suggests that while strain selection is important, the temporal dynamics of the intervention and the use of multi-strain formulations play a decisive role in clinical outcomes. The identification of an 8–24 week window for peak efficacy suggests that future research should strictly adhere to evidence-based treatment durations to avoid premature termination of efficacy. Additionally, large-scale, long-term clinical trials are essential to validate the efficacy and safety of these functional foods in MASLD treatment. Ultimately, combining probiotics and prebiotics with conventional therapies may offer a more holistic approach to managing MASLD and its associated metabolic disorders.

## Data Availability

The original contributions presented in this study are included in the article/supplementary material, further inquiries can be directed to the corresponding author.
